# Lessons from the first report of the Arrhythmogenic Right Ventricular Cardiomyopathy Registry of South Africa

**DOI:** 10.5830/CVJA-2010-037

**Published:** 2010-06

**Authors:** Neil Hendricks, David A Watkins, Bongani M Mayosi

**Affiliations:** Department of Medicine, Groote Schuur Hospital and University of Cape Town, South Africa; Department of Medicine, Groote Schuur Hospital and University of Cape Town, South Africa; Department of Medicine, Groote Schuur Hospital and University of Cape Town, South Africa

The Arrhythmogenic Right Ventricular Cardiomyopathy (ARVC) Registry of South Africa was established in 2004, under the auspices of the Cardiac Arrhythmia Society of Southern Africa (CASSA).^1^ In the November 2009 supplement of *Heart Rhythm*, the Registry investigators published the first large, multicentre series of patients with ARVC in South Africa.^2^ The report, which is based on the first 50 participants with a confirmed diagnosis of ARVC, has practical implications for the clinical profile, diagnosis and management of patients with ARVC in South Africa.

What is the profile of the patient with ARVC in South Africa? The disease occurs in all racial and ethnic groups. It usually starts causing symptoms by the third decade of life. Males manifest with symptoms of the disease more often than females, with a 2:1 ratio. Most frequently, patients come to their physician complaining of palpitations, dizziness and syncope. About a third of South African patients also notice chest pain, which makes ARVC an important consideration for clinicians evaluating young people with chest pain.

These young people with ARVC are regularly involved in sport, and in fact, 28% of patients in the South African cohort were professional endurance athletes at some point in their lives. Previous human and animal studies have demonstrated that, if an individual is genetically prone to ARVC, endurance exercise is an important environmental factor in determining how quickly the condition develops.^3^,^4^ The high proportion of sport participants in this report reiterates the need for physicians to screen endurance athletes for heart disease, both pre-participation and throughout their sporting careers.

Diagnosing ARVC can be challenging, though. Standard cardiology tests can be unremarkable for years during the earlier phases of ARVC, which means that a high index of suspicion and repeat evaluations, with close follow up, are needed. Even in the South African cohort, which typically represented advanced disease, 6% of subjects had a ‘normal’ resting ECG at their last follow-up visit. Some 12% of symptomatic patients had no abnormalities at all on cardiac imaging and were diagnosed based on family history, electrocardiographic abnormalities and molecular genetic testing.

Make no mistake, however, this is not a benign cardiomyopathy. Several Registry participants had died by the end of the follow-up period (a median of around eight years). The annual mortality in this study was 2.8%, and the five-year cumulative mortality was 10%. Outcomes were worse than in other parts of the world; on average, patients died two decades earlier than patients in France.^5^ There are several explanations for this, the most likely of which is that implantable cardioverter-defibrillators (ICDs), which are life-saving for ARVC patients, are underutilised in South Africa. What is more, for the reasons discussed above, patients do not always find their way to an expert who can diagnose and manage this complex disease.

As a striking demonstration of the severity of ARVC, outcomes were compared with control patients who represented the general South African population. The two groups had the same survival rates. Bear in mind that South Africa is in the midst of an unprecedented increase in mortality, due in large part to the colliding epidemics of HIV/AIDS and non-communicable diseases.^6^ It is not a surprise that survival from ARVC, a potentially lethal disease, is similar to survival after the combined effects of HIV/AIDS and other diseases have taken their toll on the population.

Despite such poor outcomes, however, there is an important silver lining to this cloud. The survival study identified the patients who were at high risk of dying suddenly, including those with a history of syncope and sustained ventricular tachycardia. Arrhythmias were the cause of death in two out of every three patients who died, although fortunately, to date not a single Registry participant who has received an implantable cardioverter defibrillator has died. From a practical standpoint, these data suggest that the early use of ICDs in patients with syncope and sustained ventricular tachycardia may prevent early mortality. Any patients with these symptoms and a diagnosis of ARVC should have top priority to be evaluated by a cardiologist.

It has been known for years that ARVC is an inherited condition in a significant proportion of cases. The Registry report has made several key observations about the genetic causes of ARVC. First of all, 30% of participants in the study had a family member who was also affected. For the purposes of the *Heart Rhythm* report, genetic analysis was focused on the most common culprit gene, known as plakophilin-2 (*PKP2*). Of the blood samples analysed, 25% had a *PKP2* gene defect that caused disease. While this detection rate is satisfactory in itself, the yield of genetic testing is likely to be even higher in the future; the laboratory team is currently incorporating other ARVC-related genes into the screening technique. All physicians who care for patients with ARVC are encouraged to send blood samples to the Cardiovascular Genetics Laboratory in the Hatter Institute for Cardiovascular Research at the University of Cape Town for genetic screening.

In the process of screening the *PKP2* gene, Registry investigators made another observation. One of the study subjects actually had two separate defects in the *PKP2* gene, and when his sister was screened, she was found to harbour the same two defects. The other family members had either one or the other defect – and in none of them was the disease particularly severe. The two siblings with multiple defects, however, had disease onset in early childhood, and its course was so severe that both siblings needed heart transplants by the time they were teenagers. Geneticists refer to this type of phenomenon as an ‘allele dose effect’, and to date, this is only the second time that such an effect has been reported in the ARVC research literature.

Perhaps the most important genetic discovery, however, was that several unrelated study participants actually had the exact same defect in their *PKP2* gene. Haplotype analysis revealed that, in all likelihood, these individuals received their defective gene from a common ancestor (founder effect). The founder effect, as is the case with the allele dose effect, represents only the second time that such an effect has been reported in the ARVC literature. What is more promising, however, is the potential for better understanding how this specific gene abnormality can cause a clinically distinct form of ARVC. Since investigators have identified identical defects in several individuals, they will be able to study the effects of this particular defect on a large scale.

To conclude, the CASSA-sponsored Registry has made important contributions to our understanding of ARVC in the South African context. It shows that ARVC has a high mortality and affects individuals in the prime of their life. Death is potentially preventable though, if the condition can be diagnosed early and high-risk individuals are promptly referred for consideration for ICD implantation. Because ARVC runs in families, it is mandatory to screen the first-degree relatives of affected individuals. Genetic testing yields an answer in one out of four cases and is available through the ARVC Registry at the University of Cape Town. Cardiologists and general physicians alike now have the chance to work together to improve the diagnosis and management of patients with ARVC, to reduce the high mortality rate associated with this condition. The ARVC Registry of South Africa is in a unique position to facilitate such advances in patient care, and doctors are encouraged to refer all suspected or confirmed cases of ARVC to the Registry coordinating centre for enrollment and evaluation.
